# Global Occurrence and Emission of Rotaviruses to Surface Waters 

**DOI:** 10.3390/pathogens4020229

**Published:** 2015-05-13

**Authors:** Nicholas M. Kiulia, Nynke Hofstra, Lucie C. Vermeulen, Maureen A. Obara, Gertjan Medema, Joan B. Rose

**Affiliations:** 1 Department of Fisheries and Wildlife, Michigan State University East Lansing, MI 48824, USA; E-Mails: maureenobara@yahoo.com (M.A.O.); rosejo@msu.edu (J.B.R.); 2 Enteric Viruses Research Group, Institute of Primate Research, P.O Box 24481, 00502 Karen, Nairobi, Kenya; 3 Environmental Systems Analysis Group, Wageningen University, P.O. Box 47, 6700 AA, Wageningen, The Netherlands; E-Mails: nynke.hofstra@wur.nl (N.H); lucie.vermeulen@wur.nl (L.C.V.); 4 Faculty of Civil Engineering and Geosciences, Delft University of Technology, P.O. Box 5048, 2600 GA, Delft, The Netherlands; E-Mail: gertjan.medema@kwrwater.nl; 5 KWR Watercycle Research Institute, Groningenhaven 7, 3433 PE, Nieuwegein, The Netherlands

**Keywords:** rotavirus, global, sanitation, feces, contamination, sewage, emission, modeling

## Abstract

Group A rotaviruses (RV) are the major cause of acute gastroenteritis in infants and young children globally. Waterborne transmission of RV and the presence of RV in water sources are of major public health importance. In this paper, we present the Global Waterborne Pathogen model for RV (GloWPa-Rota model) to estimate the global distribution of RV emissions to surface water. To our knowledge, this is the first model to do so. We review the literature to estimate three RV specific variables for the model: incidence, excretion rate and removal during wastewater treatment. We estimate total global RV emissions to be 2 × 10^18^ viral particles/grid/year, of which 87% is produced by the urban population. Hotspot regions with high RV emissions are urban areas in densely populated parts of the world, such as Bangladesh and Nigeria, while low emissions are found in rural areas in North Russia and the Australian desert. Even for industrialized regions with high population density and without tertiary treatment, such as the UK, substantial emissions are estimated. Modeling exercises like the one presented in this paper provide unique opportunities to further study these emissions to surface water, their sources and scenarios for improved management.

## 1. Introduction

Global estimates have reported that in 2010, 1.8 billion people drank unsafe water and an additional 1.2 billion people were exposed to drinking water with substantial sanitary risk [1]. As of 2004, approximately 2.6 billion people lacked adequate sanitation [2,3] and this figure has seen little improvement [4,5,6]. The combination of inadequate sanitation, poor hygiene, and unsafe drinking water [7] is responsible for an estimated annual burden of 0.7 million diarrheal deaths [8], making diarrhea the fourth leading cause of death among children under five years of age [9]. The Millennium Development Goal (MDG) 7, Target 7.C was to “halve, by 2015, the proportion of the population without sustainable access to safe drinking water and basic sanitation” [6,10]. While the goal for access to improved sources of drinking water has been met, the goal for proper sanitation has not been achieved. Even with access to improved water and sanitation sources, the safety of the water for domestic use is not always guaranteed [6,11].

Water quality will remain an important issue as the Sustainability Development Goals (SDGs) are developed to address the post 2015 targets. For sewage and sanitation, several targets (2015–2030) have been articulated to reduce open defecation and improve treatment. These include:

1. “All urban wastewater is adequately treated before being reused or discharged to the (aquatic) environment.
2. All wastewater is managed in a sustainable way to protect water resources and aquatic ecosystems.” [12].

Achieving the SDGs is important for reducing waterborne diseases. Rotavirus (RV) epitomizes these waterborne diseases as the enteric viruses are specific to human sewage and represent the impact that poor sanitation has on health [13]. Rotavirus was first identified in humans in the 1970s via electron microscopic examination of the duodenums of children who had severe diarrhea [14] and is a uniquely segmented, double stranded ribonucleic acid (RNA), non-enveloped virus in the family *Reoviridae* [15]. Rotaviruses are classified into at least seven serogroups, A–G, on the basis of distinctive antigenic and genetic properties [15]. Group A, B and C RVs are known to cause disease in humans [15] and group A RVs are the most important cause of acute gastroenteritis in infants and children globally [16].

The presence of RV in water sources is a public health concern due to its impacts on young children, low infectious dose (dose-response models demonstrate that RV is one of the most infectious pathogens, ID_50_ is ~6 viral units) [17], persistence, and excretion rates [18,19,20]. In infected people, RVs are excreted in very large quantities; >10^10^–10^12^ per gram and/or milliliter of stool are reported [19,21].

The primary mode of transmission of RV is the fecal-oral route via person-to-person contact or ingestion of fecally contaminated food and water, with waterborne being one of the important exposure pathways [22,23,24]. In young children, the onset of symptoms is sudden and includes moderate to severe watery diarrhea, vomiting, fever, abdominal discomfort and dehydration [21,25]. The RV incubation period ranges from 5–7 days but it may be as short as 48 h [21,26]. Thus, once the virus enters a community due to contaminated water it can spread quickly through other fecal oral routes.

The virus can also affect older children and adults but the severity of the disease is highest in children below two years of age [16,27,28,29,30,31]. As of 2008, the global RV surveillance network coordinated by World Health Organization (WHO) estimated an annual global mortality as a result of RV infections of approximately 453,000 (range 420,000 to 494,000) [32]. Rotavirus accounted for about 5% of all deaths in children and a cause-specific mortality rate of 86 deaths per 100,000 population of those below 5 of years of age [16,32]. Rotavirus has also been reported as the most common cause of severe and fatal diarrhea and it is associated with 28% of severe cases and 28% of fatal cases before vaccination was introduced [8]. The severity of the infection and the ability of RV to spread quickly (and via water) has promoted an interest in understanding the global discharges from feces and sewage into the water environment, in order to more effectively combat RV transmission via the water route.

Although water was previously considered to be less important for RV transmission compared to person-to-person contact [33], the true attribution of community cases is not well documented. Many cases of RV-associated waterborne disease have been reported in several countries, such as Greece [34], France [35], USA [36], Turkey [37], Albania [38] and Italy [39].

Rotavirus contamination of water is widespread and is demonstrated by its detection in sewage and water sources in several studies. For instance, in Kenya, clinically relevant RVs were identified in rural and urban Kenya water sources [40]; and in South Africa, RV was detected in water sources and raw vegetables [41]. In France, RV was detected in drinking water from homes of children who had been diagnosed with acute gastroenteritis caused by RV [42]. In the Netherlands, RVs have been detected in a water source reservoir and rivers [43]. Sewage and receiving surface water may be an environmental reservoir and a transmission route [17]. Therefore, RV monitoring in water sources that are used for domestic, irrigation and recreation purposes has become important to assess the public health risks of RV [44,45].

Improved hygiene, water quality and sanitation practices are necessary for the prevention of diarrheal disease caused by numerous etiological agents. However, as RV is excreted in high numbers, is persistent and highly infectious, routine wastewater treatment is often not efficient enough to prevent RV transmission, yet this has not been well documented. An RV vaccine has been developed [46] to reduce the number of severe cases of the disease, hospitalizations and deaths [32]. The RV vaccine have been shown to improve the health and well-being of children in countries where it has been introduced [47,48,49,50], leading to rapid and substantial drops in hospitalization and deaths due to RV infections [51,52,53]. Although the immunity induced by the vaccine protects against severe RV disease, re-infections by different genotypes of RV can occur throughout life as these viruses have a high genetic diversity [54,55,56]. This is a concern as several studies have reported the emergence of new RV strains that are not covered by the vaccines. It also implies that environmental circulation of RV continues in vaccinated populations. Environmental monitoring of RV, including molecular characterization of RV genotypes, can serve to detect the circulation of new RV strains in the population.

Monitoring enteric pathogens in sewage systems is one of the approaches to assess the presence of pathogens that are circulating in a specific community. Infectious RVs can be discharged into water sources used for domestic, irrigation and recreation purposes such as rivers, reservoirs, seawater and lakes, due to their resistance to typical wastewater treatment processes [57]. Rotavirus monitoring data are poor and cannot provide information on the spatial distribution and hotspots associated with RV discharges globally. Modeling approaches show great promise for understanding the impacts of (partially and inadequately) treated wastewater discharges on water resources and guiding future decision-making, both in regard to the SDGs for sanitation and vaccination programs.

Many modeling approaches exist. We are interested in understanding the global distribution of potential viral water pollution associated with a lack of sanitation. Therefore, we choose to apply an update of the modeling approach that Hofstra *et al.* [58] used for *Cryptosporidium* to RV. This model estimates pathogen emissions to surface water. In the case of group A RV, only human emissions are relevant. These emissions depend on the population size, their age distribution of the population, RV incidence and RV excretion, the sanitation type and wastewater treatment based on data from the year 2010. The model outcome is RV emissions at a 0.5 × 0.5 degree latitude × longitude grid and can be used to understand spatial distribution, hotspots and main sources of emissions. The great advantage of this modeling approach is that we can get a better understanding of the situation in areas where no monitoring data exist, but where we do have model input data available. Hotspot areas can then be selected for investigative monitoring programs. Moreover, the model can be used to explore the impact of sanitation options on environmental RV emissions.

The main aim of this paper is to develop a framework, which can be used to examine the global emission of RV from human wastewater to surface waters and ultimately for supporting risk mitigation. We present a literature review to determine the three RV specific model parameters: RV incidence, excretion rates and removal by wastewater treatment (Section 2.1), a map of RV emissions to surface water worldwide (Section 2.2) calculated with the GloWPa model adapted for RV (GloWPa-Rota model version H1, Section 4.2) and discuss the use of such maps for risk mitigation and decisions on wastewater treatment to address pathogen control.

## 2. Results

### 2.1. Literature Review

In this Section we present the results of the literature review to obtain the RV specific parameters for the GloWPa-Rota model (Section 4.2). These parameters are the incidence (Section 2.1.1), the excretion rate of the population (Section 2.1.2) and the removal by wastewater treatment (Section 2.1.3). The literature review methodology is explained in Section 4.1.

#### 2.1.1. RV Incidence

We reviewed the literature on diarrheal illness and the detection of RV in clinical samples from 2000–2014. We found that 27 countries had conducted RV surveillance studies covering a period of either one or two full calendar years, and a total of 34,972 children <15 years of age with acute gastroenteritis (Table 1). Most of the studies surveyed hospitalized, and hence severe, cases of diarrhea. In these populations the mean detection rate of RV was 33.4% (range 6%–56.3%) and the median was 34.3%.

Overall, the majority of RV infections studied occurred in children under five years of age and the rate of RV detection varied somewhat between countries (Table 1). Among the 27 studies that we included in our review, three countries reported enrollment of children below 15 years. These counties were Argentina, France and Tunisia with a RV detection rate that was between 19.7%–48.8%. One country (Saudi Arabia) recruited children under six years of age and RV accounted for 6% of all diarrheal cases. The other 23 enrolled only children below five years of age and had a mean RV detection rate of 34.7% (range 19.2%–56.3%) (Table 1). In all these studies the duration of RV diarrhea was reported to be less than seven days.

**Table 1:** Summary of the rate of rotavirus infection in children with (severe) cases of diarrhea in different countries 2002–2014.

Continent	Country	Duration of Study	# Sample Recruited	Age (years)	(%) Prevalence of RV Diarrheal Cases	Reference
Africa	Kenya	2009–2011	500	<5	38	[59]
	Libya	2007–2008	1090	<5	31.5	[60]
	Morocco	2006–2009	1388	<5	41.7	[61]
	Morocco	2011	335	<5	26.6	[61]
	Sierra Leone	2005	128	<3	37.5	[62]
	South Africa	2003–2006	3191	<5	22.8	[63]
	Tunisia	2007–2010	435	<13	27.6	[64]
Asia	Cambodia	2005–2007	2281	<5	56	[65]
	China	2008–2009	766	<5	27.94	[66]
	China	2011–2012	767	<5	34.3	[67]
	India	2004–2008	412	<3	19.2	[68]
	India	2009–2011	1807	<5	35.9	[69]
	India	2009–2012	1191	<5	39	[70]
	India	2007–2012	756	<5	38.4	[71]
	Lao PDR	2005–2007	1158	<5	54	[72]
	Myanmar	2004–2005	2179	<5	56.3	[73]
	South Korea	2005–2007	6057	<5	22	[74]
	South Korea	2007–2008	702	<1	25.2	[75]
	Taiwan	2005–2007	3435	<5	25	[76]
Europe	Albania	2007–2010	1066	<5	21	[77]
	France	2001–2004	457	<15	48.8	[78]
	Spain	2006–2008	2048	<5	40.1	[79]
Middle East	Iran	2009–2010	163	<5	46.02	[80]
	Israel	2007–2008	472	<5	39.1	[81]
	Saudi Arabia	2002–2003	1000	<6	6	[82]
South America	Argentina	2004–2007	710	<15	19.7	[83]
	Venezuela	2013	480	<5	21	[84]

The reported detection rate of RV in clinical samples in this case is not a true reflection of the total incidence of RV infection in the entire population, particularly for RV since the detection rate reported is mostly from severe diarrheal cases and it is an estimate for children under five years of age, but not for other age groups. Additionally, people suffering from RV diarrhea may require hospitalization and emergency doctor visit more often than people with diarrhea from other pathogens. Therefore, these clinical data from selected subpopulations with severe symptoms are only a fraction of the RV circulation in the population. Alternatively, Walker *et al.* [8,85] estimated diarrheal incidence rates for children under five in different regions of the world of 2.2–4.0 episodes per child per year, also from severe episodes. A recent study on the burden and etiology of diarrheal disease in infants and young children in developing countries (the Global Enteric Multicenter Study, GEMS) estimated the incidence of RV cases for children under five with moderate to severe diarrhea to be 0.035–0.413 episodes per person per year [86]. Rotavirus incidence for the total population have been reported for Brazil, China, the Netherlands and the USA in population-based surveys and ranged from 0.0093–0.24 per year (Table 3).

For use in the model, we selected an incidence of 0.24 episodes of RV diarrhea per person per year for children under five in developing countries with Human Development Index (HDI) lower than 0.785, which is the RV incidence for children under five in Brazil and in the mid-range of the RV incidence reported in the GEMS study. For children under five in industrial countries we used a RV incidence of 0.08 based on the data for this age group from the population study in the Netherlands [87]. For the rest of the population we assumed a RV incidence of 0.01, based on the data for the under five age groups in the Netherlands [87].

#### 2.1.2. Excretion Rate

In addition to the incidence we required the excretion rate for RV cases. Data on RV excretion by symptomatic and asymptomatic children and adults are limited. Many studies indicate that RV is excreted in very large quantities (10^10^–10^12^ per gram) [19,21]. Recent studies with quantitative polymerase chain reaction (qPCR) determined the shedding of RV over time [88], but present the data in qPCR-cycles rather than in virus particles or genome copies. Moreover, studies on virus excretion by mild or asymptomatic RV cases are even more limited. 

The population excretion rate may also be estimated via sewage surveillance. Surveillance data of RV in sewage reflect RV shedding in the community and has been used to study the circulation of RV and other enteric viruses in the population [89,90,91,92,93,94,95]. Where sewage surveillance produces quantitative data on virus concentration (dynamics) in sewage, these data can be used to estimate RV shedding rates in the population. This includes all RV shedding by symptomatic and asymptomatic children and adults. We reviewed 17 studies over the last seven years (2008–2014) reporting on occurrence and/or detection of RV in untreated sewage: three studies in Africa, three in Asia, four in Europe, six in the Americas and one in the Middle East. In these studies the mean RV detection rate was 60% (range 8.3%–100%) (Table 2). In Africa (Kenya, Tunisia and Egypt), the rate of RV detection was between 8.3% and 69.2% (Table 2). In Asia, the occurrence of RV in untreated sewage was reported in China at 44.4% and in India a detection rate of 77% was reported. The four studies in Europe reported a RV detection rate of between 37.5% and 100% (Table 2). In the Americas, the occurrence of RV in untreated sewage was reported in three studies from Brazil with a mean of 58% (range 28.6%–100%); Venezuela at 66.7%, Argentina at 100% and the USA at 58.3% (Table 2). Most studies used qPCR to detect RV in sewage. Only three of the studies reported concentrations of RV in sewage. These concentrations ranged from 3.12 × 10^3^ to 2.8 × 10^6^ genome copies per liter.

**Table 2:** Summary of the occurrence of rotavirus in untreated sewage from selected countries in Africa, Asia, Europe, Middle East and Americas from the last decade.

Continent	Country	Year of Study	# Samples Collected (n)	# (%) RV Positive	RV Concentration	Reference
Africa	Egypt	2006–2007	72	6 (8.3)	NR^a^	[94]
	Kenya	2007–2008	13	9 (69.2)	NR	[40]
	Tunisia	2003–2007	125	53(42.4)	NR	[96]
Americas	Argentina	2009	52	52 (100)	NR	[97]
	Brazil	2004–2005	24	11(45.8)	NR	[98]
	Brazil	2009–2010	24	24 (100)	2.40E+05 genome copies/L ^b^	[99]
	Brazil	2009	7	2 (28.6)	NR	[100]
	USA	2011–2012	24	14 (58.3)	2.8E+06 genome copies/L ^c^	[101]
	Venezuela	2007–2008	12	8 (66.7)	NR	[102]
Asia	China	2006–2007	10	10 (100)	NR	[103]
	China	2006–2007	36	16 (44.4)	3.12E+03 genome copies/L ^b^	[104]
	India	2009–2010	144	111(77)	NR	[105]
Europe	France	2003–2004	29	11(37.9)	NR	[106]
	Italy	2006–2007	16	6 (37.5)	NR	[107]
	Italy	2010–2011	285	172 (60.4)	NR	[108]
	Sweden	2013	7	7 (100)	NR	[109]
Middle East	Iran	2010–2011	15	5 (33.3)	NR	[110]

^a^ NR—not reported; ^b^ mean concentration, ^c^ Maximum concentration.

To be able to combine sewage concentration data with data on incidence of RV cases in the population, we selected the studies with quantitative data on RV in sewage (adding one study from before the literature review selection period for the Netherlands) and combined these with data on incidence of RV in the general population.

A study in the USA reported the detection of viruses at two wastewater treatment plants in Arizona [101]. In this study samples from sewage were collected monthly for a period of one year. The concentration of several enteric viruses was determined, including group A RV, by qPCR. Rotavirus concentrations were relatively high from January–June and it was detected at lower rates in the other months, reflecting an apparent seasonality of RV diarrhea in the population. RV shedding was assumed to occur in the months in which high RV concentrations were detected in sewage (January to June, a duration of six months). The incidence of RV in the USA in the pre-vaccine years was reported as 2.7 million cases in a total population of approximately 290 million (0.93%) [111]. Virus shedding by people with RV infection is high in the first days of the illness, coinciding with the diarrheal symptoms and drops to low shedding rates that may last as long as 51 days [88]. We only considered the initial high shedding phase of seven days, since the shedding rate in the later stage is several logs lower. Using the wastewater production of 265 liters per capita per day [112], we estimated a RV shedding rate of 7.4 × 10^10^ genome copies per RV case per day (shedding rate = concentration in sewage (genome copies per liter) × wastewater per capita (liter per person per day)/((RV incidence (episodes per person per year) × high shedding duration (days))/(seasonality (months per year) × days in a month (days per month)), Table 3).

Similarly, RV shedding estimates were calculated for the Netherlands, using qPCR data from sewage [90], wastewater use [113] and a RV incidence based on a population study on diarrheal incidence and the percentage of RV detected in these diarrheal cases [87]. Again, a similar approach was used for China. A population-based surveillance study in Zhengding produced a RV incidence of 0.151 in children under five [114] and we assumed this reflected the largest part of the incidence of RV infections. Sewage concentrations of RV were reported for three wastewater treatment plants [104]. We selected the plant with the highest mean RV concentration. These data were completed with data on wastewater use in Beijing [115] to calculate the shedding. Also, in the Netherlands and China, seasonality was observed in the measured sewage concentrations, with the reported sewage concentrations found in six months of the year. To calculate the shedding, we used a seasonality correction. For Brazil, RV concentrations in sewage and wastewater use were available for Rio de Janeiro [99] together with a national estimate of the number of RV cases in young children [116] and we assumed this reflected the majority of the RV incidence in Brazil (Table 3).

The RV shedding rates that were deducted from the concentrations in sewage ranged 2 × 10^9^–7 × 10^10^ genome copies per case per day, primarily determined by the concentration that was detected in sewage (Table 3). We therefore decided to use the excretion rate of 10^10^ genome copies per case per day, together with the assumption that cases shed seven days, so 7 × 10^10^ genome copies per case per episode.

#### 2.1.3. Removal during Wastewater Treatment

The removal of viruses in wastewater treatment is important to reduce the concentrations of infectious viral particles in the water environment. Removal efficiency is variable depending on the design and operation of the treatment facility. A number of studies, primarily in industrialized countries, have characterized the rate of enteric virus removal in wastewater treatment (Table 4) but only a few studies have focused on RV removal. Wastewater treatment processes tested for virus removal include primary sedimentation, secondary biological treatment such as activated sludge and trickling filters and tertiary treatment processes, such as coagulative precipitation and sand filtration, ultraviolet (UV) light and chlorination for disinfection and membrane bioreactors. A study in China [117] analyzed RV removal and inactivation with cell culture PCR by three different wastewater treatment plants. Activated sludge treatment produced a mean log_10_ removal of range 2–2.83 and coagulative precipitation a mean log_10_ removal of 0.72 ± 0.08. More data are available on removal of other enteric viruses, for example human adenovirus (HAdV), which is prevalent in sewage.

In general, primary sedimentation does little to reduce virus concentrations. Both activated sludge and chlorination were utilized in the USA study. The virus removal by secondary treatment (with chlorination) varied between the different virus types between a mean log_10_ removal of 0.7 to 2.56. Viruses were assayed with qPCR, which may overestimate the concentration of infectious virus particles [101]. In general, secondary biological treatment reduces the concentration of all viruses. The removal ranges from around 0.8–2.5 log.

**Table 3:** Deduction of rotavirus shedding rates in the population from sewage surveillance data.

Country	Concentration in Sewage (Genome copies/L)	Seasonality in Sewage (m/y)	Wastewater per Capita (L per day)	RV Incidence (Episodes per person per year)	Shedding (Genome Copies per case per day)	References
Brazil	1.0E+06	12	144	0.24	3.1E+10	[88,99,116]
China	6.8E+03	6	190	0.15	2.2E+08	[88,104,114,115]
NL	4.6E+03	6	306	0.021	1.8E+09	[87,88,90,113]
USA	1.0E+05	6	265	0.0093	7.4E+10	[88,101,111,112]

Tertiary treatment may be comprised of very different treatment processes. The studies indicate that tertiary disinfection with chlorine or UV has limited effect on virus concentrations in practice, even where infectivity assays were used. Advanced tertiary treatment processes such as membrane bioreactors can produce a very significant reduction of virus concentrations in wastewater. Though secondary and tertiary systems are common processes for larger wastewater treatment plants in industrialized countries, wastewater treatment ponds are the most common treatment process worldwide. A recent review of wastewater treatment ponds [118] showed that virus removal in pond systems is also highly variable. In 62% of the field studies of pond systems, virus removal was more than 1 log. The authors showed that there is a weak to moderate relation between virus removal and hydraulic retention time with 1 log virus removal for every 14.9–20.7 days retention time. 

**Table 4:** Log removals of viruses by secondary and tertiary treatments.

Virus ^a^	Country ^b^	Detection Method ^c^	Treatment process(es) ^d^	n	Removal/Inactivation		Reference
					Mean (log10)	Stdev (Log10)	
RV	China	ICC-qPCR	Activated sludge	12	2.08	0.63	[117]
RV	China	ICC-qPCR	Activated sludge	12	2.83	0.49	[117]
RV	China	ICC-qPCR	Activated sludge	12	2	1.1	[117]
RV	China	ICC- qPCR	Coagulative precipitation and sand filtration	12	0.72	0.08	[117]
PPMV	USA	qPCR	Activated sludge + Cl2	12	0.76	0.53	[101]
PPMV	USA	qPCR	Trickling filter + Cl2	12	0.99	0.12	[101]
AdV	USA	qPCR	Activated sludge + Cl2	12	0.7		[101]
AdV	USA	qPCR	Trickling filter + Cl2	12	1.5		[101]
JCPyV	USA	qPCR	Activated sludge + Cl2	12	1.64	0.98	[101]
JCPyV	USA	qPCR	Trickling filter + Cl2	12	2.56	0.64	[101]
BKPyV	USA	qPCR	Activated sludge + Cl2	12	1		[101]
BKPyV	USA	qPCR	Trickling filter + Cl2	12	1.5		[101]
AiV	USA	qPCR	Activated sludge + Cl2	12	0.94	0.33	[101]
AiV	USA	qPCR	Trickling filter + Cl2	12	0.99	0.12	[101]
EV	USA	qPCR	Activated sludge + Cl2	12	1.5		[101]
EV	USA	qPCR	Trickling filter + Cl2	12	2.1		[101]
EV	NL	CC	Activated sludge	5	1.4	0.42	[90]
ReoV	NL	CC	Activated sludge	5	1.2	0.22	[90]
HAdV	Spain	qPCR	Tertiary		1.2		[119]
HAdV	Spain	qPCR	Tertiary		1.9		[119]
AdV	USA	qPCR	Membrane Bioreactor		3.9–5.5		[120]
NoVII	USA	qPCR	Membrane Bioreactor		4.6–5.7		[120]

^a^ RV—rotavirus, PPMV—pepper mild mottle virus, HAdV—human adenovirus, JCPyV—JC polyomavirus, BKPyV—BK polyomavirus, AiV—aichivirus, EV—enterovirus, ReoV—reovirus, NoVII—norovirus genogroup 2; ^b^ USA—United States, NL—Netherlands; ^c^ ICC-qPCR—integrated cell culture and quantitative polymerase chain reaction, CC—cell cultures; ^d^ Cl2—chlorination.

For the model, we have assigned 0.1 log virus removal to primary sedimentation, 1.5 log virus removal to secondary biological treatment and 0.5 log to tertiary treatment. This corresponds to 20% removal for primary treatment, 97.5% removal for primary followed by secondary treatment and 99.21% removal when tertiary treatment is added. Lagoons and other tertiary treatment methods may have different removal capacities, but these were not reported within the available treatment datasets.

### 2.2. Global Rotavirus Emissions

**Figure 1:**
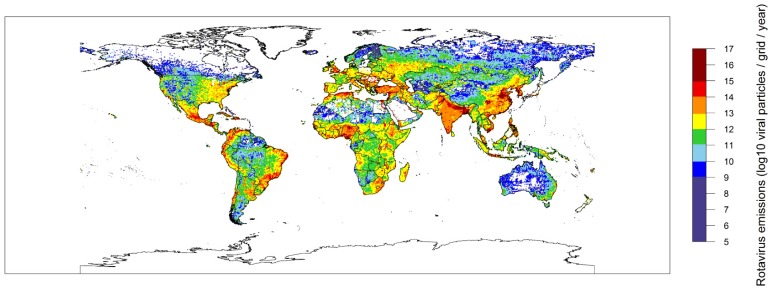
Total RV emissions in log_10_ viral particles per grid (based on data for approximately the year 2010).

We have used the excretion rates and removal fractions distilled from the literature in Section 2.1.1 to develop the GloWPa-Rota model. This model is explained in detail in Section 4.2. The model was used to produce an emission map (Figure 1). This map shows the global distribution of human RV emissions per 0.5 × 0.5 latitude × longitude grid (based on 2010 data for population, access to sanitation and waste water treatment (none, primary, primary + secondary, or primary + secondary + tertiary). Total global RV emissions are 2 × 10^18^ viral particles/grid/year, of which 87% is produced by the urban population. Urban areas also stand out in Figure 1. High emissions are observed in urban areas in Bangladesh, East China, India, Korea, Turkey, Nigeria, the northern part of Algeria, the UK, Belgium, Mexico, Colombia and coastal Brazil. Low emissions are observed in rural areas in Canada, Scandinavia, Russia, China, the Sahel and Australia. Main drivers for the spatial differences in emissions are population size in a grid, sanitation type and removal by wastewater treatment plants.

We zoom in on two example countries to get an understanding of the spatial differences. We picked Nigeria as an example of a country with high concentrations of RV emissions and the United Kingdom (UK) as an example of an industrialized country with relatively high emissions. Total emissions in Nigeria are 1.1 × 10^17^ and in the UK 8.9 × 10^15^. For Nigeria 18% and for the UK 13% of total emissions by the population reach the surface water. The population of Nigeria (158 million people, 50% urban) is much larger than that of the UK (62 million, 78% urban) and the sanitation types are very different. The sanitation types are summarized in the categories ‘connected to sewer’, ‘direct emissions’, ‘diffuse emissions’ and ‘non source’ as explained in Section 4.2. In urban areas in Nigeria, only 18.8% of the population is connected to a sewer and there is no treatment available. In rural areas, only 3.8% of the population is connected to a sewer without treatment [121]. In total, 24.7% of the urban and 3.3% of the rural population emit directly into surface water. In rural areas, 39.8% of the people produce diffuse emissions. The remaining people in Nigeria have access to septic tanks or pit latrines and we assume their emissions do not enter the surface water (non-source). In the UK in urban areas 100% of the population is connected to a sewer system and in rural areas 73% (the remaining 27% is a non-source) and they have access to no (9%), primary (9%), primary + secondary (56%) or primary + secondary + tertiary (26%) treatment, respectively.

**Figure 2:**
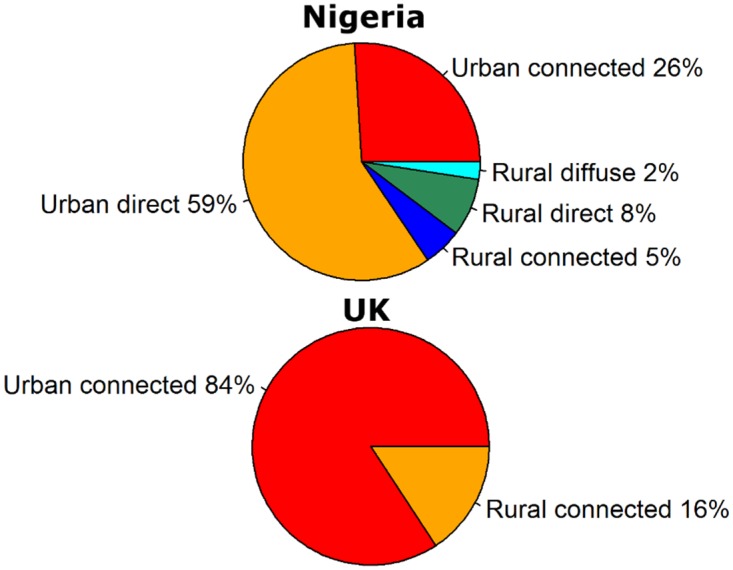
Fraction of the emissions caused by population with access to the different sanitation types for Nigeria (**top**) and UK (**bottom**).

The difference in access to sanitation types is immediately visible when looking at the fraction of emissions caused by people with access to the different sanitation types (Figure 2). In both regions, the majority of the emissions comes from urban areas (85% in Nigeria; 84% in the UK). In Nigeria, 59% are direct emissions, but since there is no treatment, also the emissions from the population connected to sewers are actually direct emissions. Rural emissions are mostly from the population connected to sewers (5% in Nigeria and 16% in the UK) and direct emissions (8% in Nigeria). Only a small part of the emissions in Nigeria (2%) are diffuse emissions. This number is relatively low; the percentage of the feces on the land that enters the surface water is assumed to be only 2.5% (see Section 4.2).

**Figure 3:**
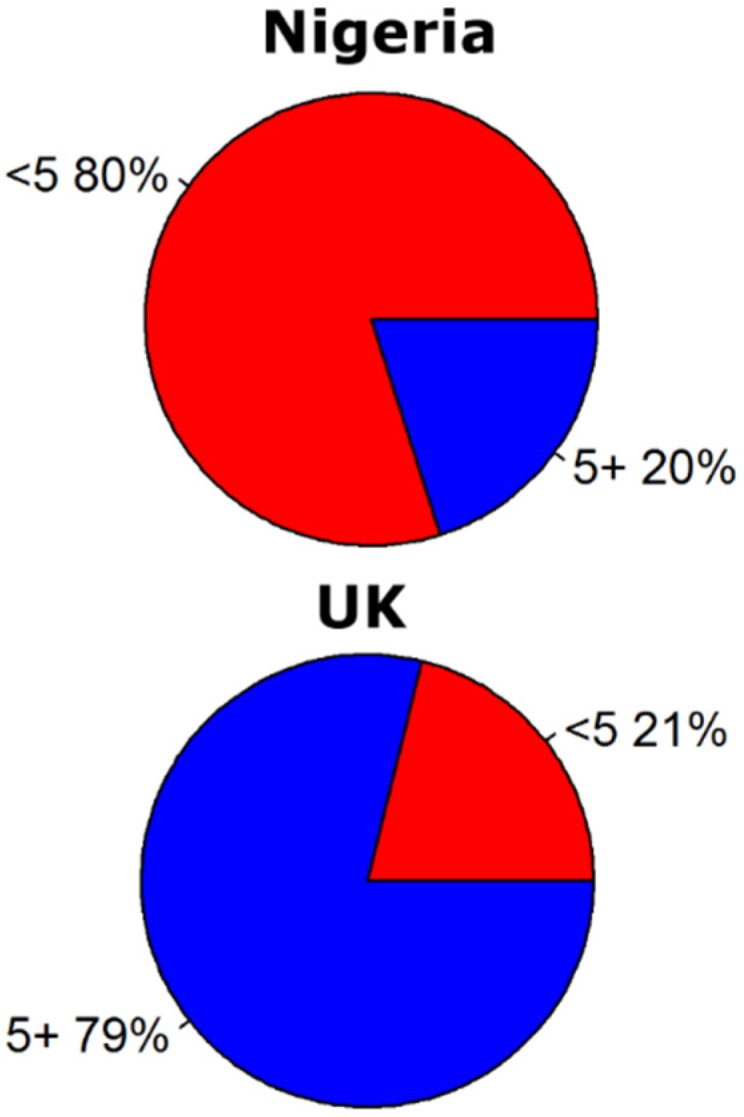
Fraction of the emissions caused by population from the age categories under five and over five for Nigeria (**top**) and the UK (**bottom**).

We have also studied the differences in emissions between age groups. Children under two years of age are more susceptible to RV and they shed much more viruses in their feces than other age groups, who often have more asymptomatic shedding. Figure 3 provides the pie charts for Nigeria and the UK. The difference between both countries is remarkable. In Nigeria the population younger than five years of age is responsible for 80% of the emissions, while in the UK the same age category only produces 21% of the emissions. The main variables driving this difference are the higher percentage of children under five years of age in Nigeria (17.5% compared to 6.3% in the UK), the RV incidence rate in children under five that is assumed to be higher in Nigeria (0.24) than in the UK (0.08) and the assumption that in the UK all children under 2.5 years of age wear nappies, while in Nigeria only the children under 2.5 years of age with access to a sewer do.

## 3. Discussion and Conclusions

To our knowledge, this is the first paper estimating the global distribution of RV emissions, which we believe will serve as an example for a number of important waterborne pathogens. There is a significant need to understand biological water quality and the concentration of pathogens in global water systems. This knowledge base is needed to create an efficient framework for achieving the new Sustainable Development Goals directed toward progress in sanitation and drinking water safety. Thirty years ago, Feachem *et al.* [122] synthesized existing scientific knowledge on the occurrence and persistence of pathogens in the environment. Since its publication, this document has been the key reference point for the development of quantitative guidance for safe drinking water and sanitation practices including defining safe and unsafe activities, evaluation of low technology treatment and disposal options, and for addressing adequate controls for protection of health. Over the past decades, there has been a dramatic increase in knowledge and data regarding emerging pathogens, new technologies for measuring pathogens, new information on pathogen occurrence and persistence, and new interventions. A global consortium of researchers in the field of health-related water microbiology are committed to updating and producing a new resource, so that the knowledge can be translated into life-saving interventions. The paper contributes to this global water pathogen project (GWPP) [123], which aims to obtain a better understanding of occurrence, fate and transport of pathogens in sewage and surface water ultimately used to define the risks associated with the emissions of pathogens to surface water from fecal wastes and wastewater. 

The RV map shows global coverage of emissions, except for some very remote areas in deserts or at high latitudes. Emissions are highest in urban areas and in most countries these account for the majority of the emissions. Reasons for this include the population density in these regions and the fact that emissions by the population not connected to sewer systems are assumed to be direct emissions to surface waters in our model. However, one of the main determining factors for the order of magnitude of the emissions calculated by our model is the excretion rate, which is estimated based on the literature. A wide shedding range is quoted (>10^10^–10^12^ per gram feces) but good data on RV shedding by symptomatic or asymptomatic children and adults are limited. The shedding rates are related to the age and distribution over the course of the infection [19,21,88]. The range was found to be too high for an average to explain the concentrations found in sewage. We therefore used a reverse calculation from sewage concentrations and infection rates to determine the excretion rate per RV illness case per year. 

Other determining variables for the emission maps are the RV incidence and the removal by wastewater treatment. We extracted these data from the literature as explained in Section 2.1. Data on RV incidence are limited and what is available shows considerable differences in incidence between study populations and countries. Similarly, for RV removal by wastewater treatment data availability is limited and virus removal rates vary between virus and treatment system. Also, the coverage data of primary, secondary or tertiary treatment in a country do not specify lagoons or disinfection. Additional data on RV incidence in the general population, RV removal by wastewater treatment and on treatment coverage would increase the robustness of the estimated emissions.

The results of our analysis show that some areas have relatively low emissions, because a large part of their population use septic tanks or pit latrines that have limited influence on surface water quality and hence were classified as a non-source in our model. That may be an underestimation, as often these tanks and latrines are emptied and their contents either treated or dumped in the surface water directly. Obviously, the excreta will have had a residence time in the tanks or latrines, reducing the RV concentrations, but still some RV may enter the surface water via this route.

The emission maps are a first step towards a better understanding of RV hotspots. In future studies, the emissions could be converted to surface water concentrations to enable risk assessment. Moreover, scenario analysis could be performed. Such an analysis could study how RV concentrations change if vaccination is applied that strongly reduces the RV incidence in children under five years of age as is indicated in the literature [49,51,53], if immunity in the population is included, or if specified sanitation strategies are implemented. Such management strategies could include improved sanitation for all, tertiary treatment that includes disinfection and other combinations of treatment improvements for different countries. Finally, the seasonality of RV surface water concentrations could be studied further. This seasonality is visible from the sewage concentrations (Table 3) and influences the treatment required to reduce the health risk.

Our analysis can be used to evaluate different sanitation scenarios also on country level. The analysis of UK and Nigeria suggests that connecting the population to sewers may even increase emissions to surface water, if the sewage is not properly treated. Connection to sewers of people that previously used a latrine or open defecation will now take RV and other pathogens to surface water. Even in the UK, where almost everyone is connected to a sewer, but where only a limited percentage of the sewage is treated by tertiary treatment, emissions are considerable. Very few countries have full coverage with tertiary treatment that includes disinfection. This would be essential to reduce RV emissions to surface water. The focus of the SDGs on ending open defecation may therefore result in increased emissions to surface water unless it is planned in conjunction with improved wastewater treatment to protect water resources.

Rotavirus is a major source of diarrheal infection worldwide. This paper provides insight in the spatial differences and hotspot areas in the RV emissions and opens the possibility of scenario analysis to evaluate alternative sanitation strategies. Hotspot areas include urban areas in densely populated parts of the world, such as Bangladesh and Nigeria, while low emissions are estimated in rural areas with low population density, such as in northern Russia or the Australian desert. Modeling exercises like the one presented in this paper provide unique opportunities to further study these emissions to surface water, their sources and scenarios for improved management.

## 4. Experimental Section 

### 4.1. Literature Review

#### 4.1.1. Search Strategy

We searched for all studies reporting the epidemiology and occurrence of RVs in pediatrics in online databases, including Google Scholar, PubMed, PubMed Central, Science Direct and the citation tracking system of related articles thorough Thomson Reuters’ Web of Science, for articles published between 2000 and 2014. For the occurrence of RV in untreated sewage, we searched for articles published from 2008 to 2014. To estimate the RV removal by wastewater treatment, we searched literature of 2005 to 2015. Only relatively recent studies were included since molecular virus detection methods in sewage have evolved rapidly over the past decade. We restricted the search to only the articles published in the English Language. We used key words/subject terms, including one or combinations of the following: “Rotavirus infection AND humans”, “diarrhea or diarrhea”, “global epidemiology of rotavirus” and/or “acute gastroenteritis OR diarrhea”, “burden of rotavirus infection”, “rotavirus in Africa AND Asia, Europe, America, and Australia”. We also used the articles identified by using the PubMed option of related articles and manually checked the reference lists of the original and review articles.

We used the same search strategy for occurrence of RV in untreated sewage using keywords like “rotavirus AND sewage”, “occurrence of rotavirus in sewage”, “surveillance of rotavirus in sewage”, “molecular characterization of rotavirus in sewage”. In most cases, we had to read the articles to identify cases where only untreated sewage or influent was reported. We also included the papers that reported concentration of RVs in sewage.

To get an understanding of the removal rates by wastewater treatment, we used the keywords “wastewater” or “sewage” and “treatment and rotavirus”. Only few papers were found that included RV and therefore we extended the search to other viruses.

#### 4.1.2. Quality Assessment

To assess the quality of the articles all the entries were checked and edited, including the clinical demographic data. Articles that were selected were assessed for quality data by looking at the data presentation. Experiences as part of the WHO RV surveillance team and facilitation of data quality assessment using WHO checklist and guidelines used for the global surveillance of RV helped to judge the assessment quality. 

### 4.2. Explanation of the GloWPa-Rota H1 Model

We estimate human emissions for RV based on the approach of Hofstra *et al.* [58] used for *Cryptosporidium*. This exploration of global *Cryptosporidium* emissions used the preliminary version of the Global Waterborne Pathogen (GloWPa) model that we present here for RV. We use the updated version of the human emissions part of the model that is in Vermeulen *et al.* [124] applied in a case study for India and Bangladesh. The updated version improves the model by including emission from people not connected to sewer systems in the model.

We present here the GloWPa-Rota H1 model (Global Waterborne Pathogen model for RV version 1 for Human emissions). Humans, through their feces, are the only source of RV. We estimated human emissions for the year 2010 based on population, sanitation availability and excretion by the population. The base equation for the calculation for a country is:

(1) $$Total\;human\;emissions\;H=P\times f_{san}\times V_{p}\times f_{sw}$$

Where *P* is the population of the country, *f_san_* the fraction of the population using a particular type of sanitation facilities (connected to sewer, direct emissions, emissions on the land), *V_p_* is the viral particles excretion rate per person, as explained in Section 2.1 and *f_sw_* the fraction that reaches the surface water (and is not removed by waste water treatment or left behind on the land). 

We estimate emissions for different age categories: 0–4 years, 5–15 years, 15–25 years and 25+ years of age, because diarrheal incidence and prevalence of RV are different for each age category:

(2) $$H=\;\overset{25+}{\underset{age=<5}{\sum }}H_{age}$$

Where *age* is the age category (<5, 5–14, 15–25 and 25+ years of age). 

We estimate emissions separately for urban and rural areas, because sanitation is different in each area. Additionally, we assume that in the industrial countries (Human Development Index (HDI) > 0.785) all children use nappies until the age of 2.5 and their feces are thrown away and therefore are not emitted in the sewage, while in developing countries (HDI < 0.785) only children under 2.5 years of age living in households that are connected to sewers wear nappies. To allow for the nappies and a lower weight of stools, the equations for H_<5_ are different from the other age categories:

(3) $$H_{<5}=\{\begin{array}{c} (CE_{u,<5}+\;CE_{r,<5}+DE_{u,<5}+DE_{r,<5}+DifE_{r,<5})/2\;if\;HDI>0.785\\ \frac{CE_{u,<5}}{2}+\frac{CE_{r,<5}}{2}+DE_{u,<5}+DE_{r,<5}+DifE_{r,<5}\;if\;HDI<0.785 \end{array}$$

(4) $$\begin{array}{c} H_{age}=CE_{u,age}+\;CE_{r,age}+DE_{u,age}+DE_{r,age}+DifE_{r,age}\;for\;age\\ =5-14,\;15-25\;and\;25+ \end{array}$$

Where *CE* are the emissions from people connected to sewer systems in urban (*u*) and rural (*r*) areas, *DE* are direct emissions from people with hanging toilets (both urban and rural) and without sanitary facilities (urban areas only). Finally, *DifE* are emissions from people defecating on the land in rural areas. We assume that emissions from people using septic tanks and pit latrines do not reach the surface water.

For each emission category, emissions are calculated as follows:

(5) $$Connected\;emissions\;urban\;{\mathrm{CE}}_{\mathrm{u,age}}\;=\;P_{u}\times f_{age}\times f_{cu}\times V_{p,age}\times \;\left(1-f_{rem}\right)$$

(6) $$Connected\;emissions\;rural\;{\mathrm{CE}}_{\mathrm{r,age}}\;=P_{r}\times \;f_{age}\times \;f_{cr}\times V_{p,age}\times \;\left(1-f_{rem}\right)$$

(7) $$Direct\;emissions\;urban\;{\mathrm{DE}}_{\mathrm{u,age}}\;=P_{u}\times \;f_{age}\times \;f_{du}\times V_{p,age}$$

(8) $$Direct\;emissions\;rural\;{\mathrm{DE}}_{\mathrm{r,age}}\;=P_{r}\times \;f_{age}\times \;f_{dr}\times V_{p,age}$$

(9) $$Diffuse\;emissions\;rural\;{\mathrm{DifE}}_{\mathrm{r,age}}\;=P_{r}\times \;f_{age}\times \;f_{difr}\times V_{p,age}\times f_{run}$$

Where:

- *P_u_* and *P_r_* are the total urban and rural population of a country, respectively.
- *f_age_* the fraction of the population for the age categories (<5, 5–14, 15–25 and 25+)(−).
- *f_cu_* and *f_cr_* are the fractions of the urban and rural populations using sanitation that is connected to a sewer system (−).
- *f_du_* and *f_dr_* are the fractions of the urban and rural populations using sanitation that is a direct source (−). As explained in Vermeulen *et al.* [124], this includes WHO/UNICEF Joint Monitoring Programme (JMP) sanitation types hanging toilets (for both urban and rural population) and no facility, bush, field, unknown, elsewhere, other unimproved (for urban population only).
- *f_difr_* is the fraction of the rural population that has no sanitation facilities and forms a diffuse source (−). This includes JMP sanitation type no facility, bush, field, unknown, elsewhere, other unimproved Vermeulen *et al.* [124].
- *f_run_* is the fraction of feces transported with runoff from land to surface water (−). *f_run_* is assumed to be 0.025, which is the median value for animal manure mobilization estimated in Ferguson *et al.* [125]. The actual RV mobilization depends on wet weather events, runoff amounts, dry weather duration, manure accumulation on the land and survival in the manure. Such an in-depth analysis is a step too far for this paper, so we assume RV mobilization is comparable to animal manure mobilization. That may be a slight overestimation.
- *V_p,age_* is the average viral particle excretion (viral particles person^−1^·year^−1^). *V_p_* differs for the age categories and is calculated as described in Section 2.1.1.
- *f_rem_* is the fraction of viral particles removed by wastewater treatment (−). *f_rem_* is calculated as described below (Equation (10).

See Table 5 for data sources of the variables used in the model.

The wastewater of the people connected to sewer systems may be treated in a wastewater treatment plant. The removal depends on the type of treatment (no treatment, primary, primary + secondary or primary +secondary + tertiary). For each country the removal is estimated as follows:

(10) $$f_{\text{rem}}=f_{p}\times RE_{p}+f_{s}\times RE_{s}+f_{t}\times RE_{t}$$

Where f_p_, f_s_ and f_t_ are the fraction of primary, secondary and tertiary wastewater treatment systems in a country, respectively (see Table 5), and *RE_p_*_,_ *RE_s_* and *RE_t_* are the removal efficiencies for primary, secondary and tertiary wastewater treatment systems, respectively (−). The removal efficiencies are estimated from the literature as explained in Section 2.1.3 at 0.2, 0.975, and 0.9921, for primary, primary + secondary and primary + secondary + tertiary treatment, respectively.

After the human emissions (H) are calculated for each country, we spatially distribute the country data over a 0.5 × 0.5 degree latitude × longitude grid. We spatially identify a country’s urban and rural population via density ranking, where the population in the most densely populated grid cells is defined as urban, based on a LandScan 2010 density map [126]. We assume that among a population defined as urban or rural, sanitation is distributed equally, proportional to the occurrence of different sanitation types. All age categories are distributed equally over the grids.

**Table 5:** Data sources.

Variable	Variable Name	Data Source
*P*	Population	[127]
*P_u_*	Urban population	Urban fraction x P
*P_r_*	Rural population	(1 − urban fraction) × P
*f_age_*	Fraction of the population younger than 5 years of age, from 5 to 14, from 15 to 25 and older than 25.	UN World Population Prospects [128]
*HDI*	Human Development Index	[129]
*f_cu_, f_cr_* *f_du_, f_dr_* *f_difr_*	Fraction connected (urban and rural) Fraction direct (urban and rural) Fraction diffuse (rural only)	WHO/UNICEF JMP data [121], www.wssinfo.org). Year closest to 2010 was taken from JMP country files. When unavailable, fractions were estimated based on the fraction connected used in Van Puijenbroek *et al.* [130], which were based on WHO/UNICEF [121] supplemented with data from [131,132,133]. When incomplete (mostly missing values only around 0.01–0.02), missing values were added to non-source, or in case non-source was non-existing, to the category with the highest fraction.
*f_p_, f_s_ and f_t_*	Fraction primary, primary + secondary and primary + secondary + tertiary treatment	[131,132,133] as explained in Van Puijenbroek *et al*. [130]
	Population density in a grid cell	LandScan 2010 data [126]
