# Correction: Kiulia, N.M. *et al.* Global Occurrence and Emission of Rotaviruses to Surface Waters. *Pathogens* 2015, *4*, 229–255

**DOI:** 10.3390/pathogens5010026

**Published:** 2016-03-01

**Authors:** Nicholas M. Kiulia, Nynke Hofstra, Lucie C. Vermeulen, Maureen A. Obara, Gertjan Medema, Joan B. Rose

**Affiliations:** 1 Department of Fisheries and Wildlife, Michigan State University East Lansing, MI 48824, USA; maureenobara@yahoo.com (M.A.O.); rosejo@msu.edu (J.B.R.); 2 Enteric Viruses Research Group, Institute of Primate Research, P.O Box 24481, 00502 Karen, Nairobi, Kenya; 3 Environmental Systems Analysis Group, Wageningen University, P.O. Box 47, 6700 AA, Wageningen, The Netherlands; nynke.hofstra@wur.nl (N.H.); lucie.vermeulen@wur.nl (L.C.V.); 4 Faculty of Civil Engineering and Geosciences, Delft University of Technology, P.O. Box 5048, 2600 GA, Delft, The Netherlands; gertjan.medema@kwrwater.nl; 5 KWR Watercycle Research Institute, Groningenhaven 7, 3433 PE, Nieuwegein, The Netherlands

The authors wish to make the following corrections to their paper [1].

Replacing “2 × 10^18^ viral particles/grid/year” in the abstract and Section 2.2 with “2 × 10^18^ viral particles/year”.

The authors would like to apologize for any inconvenience caused.

The manuscript will be updated and the original will remain available on the article webpage.
